# Quantifying the fitness cost of HIV-1 drug resistance mutations through phylodynamics

**DOI:** 10.1371/journal.ppat.1006895

**Published:** 2018-02-20

**Authors:** Denise Kühnert, Roger Kouyos, George Shirreff, Jūlija Pečerska, Alexandra U. Scherrer, Jürg Böni, Sabine Yerly, Thomas Klimkait, Vincent Aubert, Huldrych F. Günthard, Tanja Stadler, Sebastian Bonhoeffer, the Swiss HIV Cohort Study

**Affiliations:** 1 Division of Infectious Diseases and Hospital Epidemiology, University Hospital Zurich, Zurich, Switzerland; 2 Institute of Medical Virology, University of Zurich, Zurich, Switzerland; 3 Institute of Integrative Biology, ETH Zurich, Zurich, Switzerland; 4 Department of Biosystems Science and Engineering, ETH Zurich, Basel, Switzerland; 5 Swiss Institute of Bioinformatics (SIB), Lausanne, Switzerland; 6 School of Medicine, Imperial College London, London, United Kingdom; 7 Laboratory of Virology, Division of Infectious Diseases, Geneva University Hospital, Geneva, Switzerland; 8 Department of Biomedicine, University of Basel, Basel, Switzerland; 9 Division of Immunology and Allergy, University Hospital Lausanne, Lausanne, Switzerland; University of California, San Francisco, UNITED STATES

## Abstract

Drug resistant HIV is a major threat to the long-term efficacy of antiretroviral treatment. Around 10% of ART-naïve patients in Europe are infected with drug-resistant HIV type 1. Hence it is important to understand the dynamics of transmitted drug resistance evolution. Thanks to routinely performed drug resistance tests, HIV sequence data is increasingly available and can be used to reconstruct the phylogenetic relationship among viral lineages. In this study we employ a phylodynamic approach to quantify the fitness costs of major resistance mutations in the Swiss HIV cohort. The viral phylogeny reflects the transmission tree, which we model using stochastic birth–death-sampling processes with two types: hosts infected by a sensitive or resistant strain. This allows quantification of fitness cost as the ratio between transmission rates of hosts infected by drug resistant strains and transmission rates of hosts infected by drug sensitive strains. The resistance mutations 41L, 67N, 70R, 184V, 210W, 215D, 215S and 219Q (nRTI-related) and 103N, 108I, 138A, 181C, 190A (NNRTI-related) in the reverse trancriptase and the 90M mutation in the protease gene are included in this study. Among the considered resistance mutations, only the 90M mutation in the protease gene was found to have significantly higher fitness than the drug sensitive strains. The following mutations associated with resistance to reverse transcriptase inhibitors were found to be less fit than the sensitive strains: 67N, 70R, 184V, 219Q. The highest posterior density intervals of the transmission ratios for the remaining resistance mutations included in this study all included 1, suggesting that these mutations do not have a significant effect on viral transmissibility within the Swiss HIV cohort. These patterns are consistent with alternative measures of the fitness cost of resistance mutations. Overall, we have developed and validated a novel phylodynamic approach to estimate the transmission fitness cost of drug resistance mutations.

## Author summary

The introduction of antiretroviral therapy (ART) has decreased mortality and morbidity rates among HIV-infected people, and improved their quality of life. In fact, the WHO states that antiretroviral therapy programmes averted an estimated 7.8 million deaths worldwide between 2000 and 2014. However, the antiretroviral regimen prescribed to a patient may be unable to control HIV infection. Factors that can contribute to treatment failure include drug resistance, drug toxicity, or poor treatment adherence. In this study we aim to understand the dynamics of transmitted drug resistance by analysing the viral sequence data that was collected for resistance testing. We present a novel approach to quantify how drug resistance impacts virus lineage transmissibility, how fast resistance mutations evolve in sensitive strains and how fast they revert back to the sensitive type. We apply our approach to the Swiss HIV cohort study, and obtain patterns of viral transmission fitness that are consistent with alternative, harder to obtain measures of fitness.

## Introduction

The emergence and subsequent spread of drug resistant human immunodeficiency virus type 1 (HIV-1) is a major threat to the long-term efficacy of antiretroviral treatment. Around 10% of antiretroviral therapy (ART)-naïve patients in Europe are infected with drug-resistant HIV-1 and transmitted drug resistance (TDR) has been associated with a higher virological failure rate during treatment [1–9]. The dynamics of TDR depend largely on the respective resistance mutation and requires quantification of their fitness cost. Estimates of fitness costs, resistance evolution and reversion rates could previously only be obtained by comparing the replication kinetics of the virus after infection of cell cultures or more complicated experimental techniques [10] or through longitudinal cohort studies [11, 12]. These methods are essential in understanding the type of fitness cost related to replication within the host. Here we are interested in a different type of viral fitness, namely the transmission fitness, which describes the success of a viral lineage in transmission between hosts.

As *pol* sequences are routinely collected from infected individuals to test for drug resistance, HIV sequence data is increasingly available. These sequences can be used to reconstruct the phylogenetic relationship among viral lineages, which is an approximation of the transmission tree. A considerable number of phylogenetic and phylodynamic approaches for the analysis of pathogen outbreaks have been developed in the last decade and have greatly contributed to a better understanding of the dynamics of HIV epidemics [13–19].

In this study we employ a phylodynamic approach to quantify the fitness costs of major resistance mutations using data from the Swiss HIV cohort study (SHCS) and the associated drug resistance database. Our approach is based on stochastic birth–death–sampling processes, which have been shown to be suitable for the modelling of epidemic processes [20]. In terms of the transmission tree a “birth” event corresponds to the infection of a new host, a “death” event corresponds to the host’s removal from the infectious pool (e.g. successful treatment). The removed host may or may not have been sampled before removal, which corresponds to the viral strain being sequenced and included in the SHCS.

**Fig 1:**
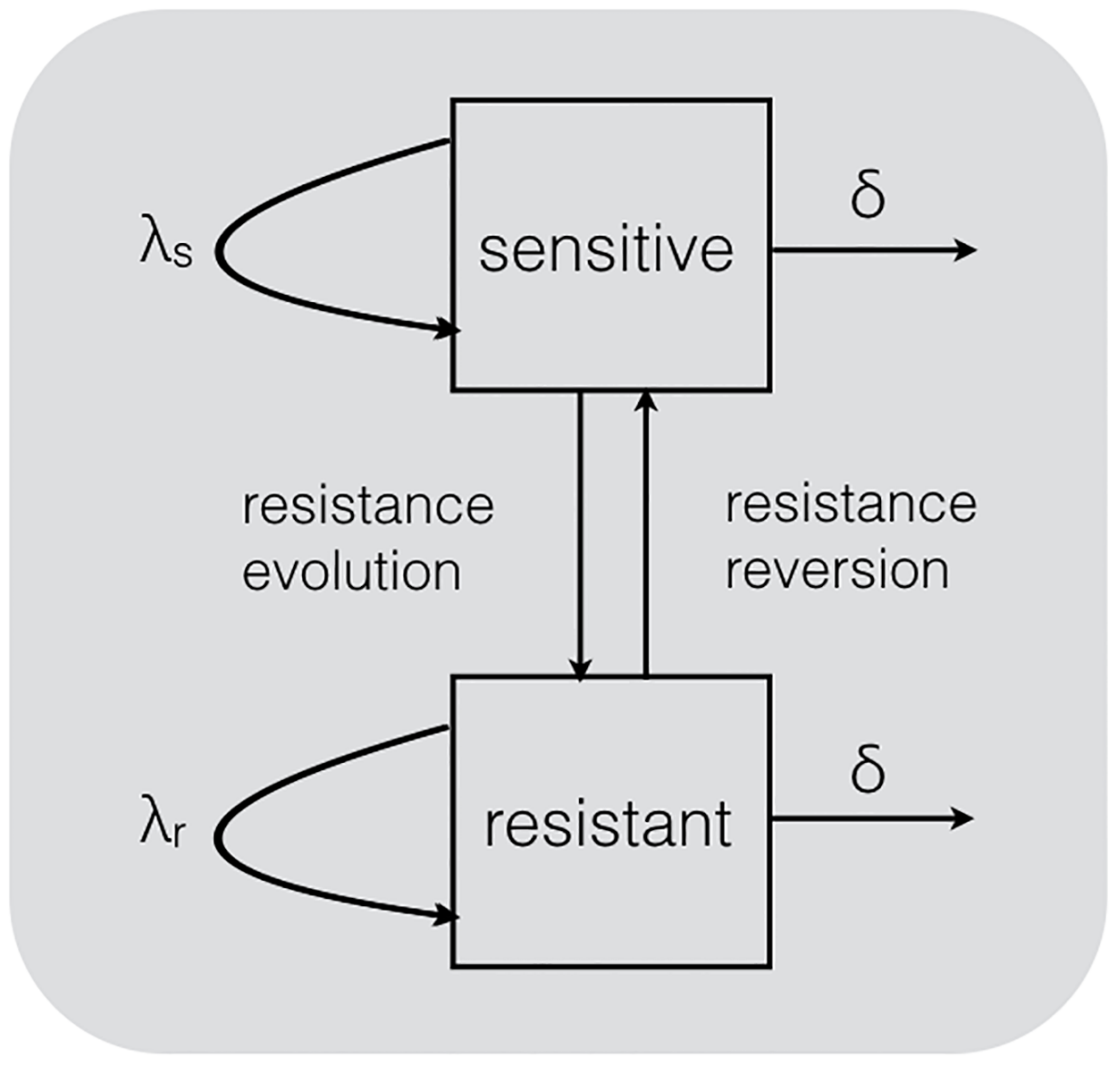
The two-type birth–death model with types ‘sensitive’ and ‘resistant’. Virus samples are grouped into the compartments by their resistance status (corresponding to a single major resistance mutation). Transmission at transmission rates λ_*s*_ and λ_*r*_ can only occur within the sensitive and resistant compartment, respectively. In either compartment, removal from the infectious pool occurs at rate *δ*. The compartments are connected by (exponential) rates of resistance evolution and reversion.

We consider each major resistance mutation separately such that our model requires exactly two types, sensitive and resistant, between which we assume a simple ‘migration’ process of resistance evolution and reversion, see Fig 1.

The aim of this paper is to demonstrate that our phylodynamic approach can shed light on the dynamics of HIV resistance evolution. In fact, the approach we present here allows us to quantify the fitness cost of major resistance mutations (i.e. λ_*r*_/λ_*s*_ in Fig 1), as well as the rates at which resistance mutations evolve and are reversed, on a population level. Notably, our approach requires only cross-sectional viral sequence data annotated with dates of sampling.

## Materials and methods

### SHCS sequence data set

The SHCS is a multicentre, prospective observational study for interdisciplinary human immunodeficiency virus (HIV) research, with an estimated coverage of more than 45% of all HIV cases in Switzerland overall and more than 70% since 1996 [21].

The associated drug resistance database is a central, anonymised collection of all genotypic drug resistance tests performed on SHCS enrolees. The base data set used in this study contains the first HIV-positive (subtype B) samples of treatment naïve SHCS patients. Due to the availability of the SHCS biobank, in total more than 12000 genotypes could be generated retrospectively, making the SHCS one of the globally most densely sampled populations [6]. In particular, all patients with available samples present before the start of ART were retrospectively sequenced. This is of particular importance because in general, HIV drug resistance testing was not routinely performed before 2003 in Switzerland or even later in other countries. Hence, the sampling dates of the sequences included here range from as early as 1989 to 2015.

The resulting 5638 *pol* sequences were combined with the 4284 most closely related sequences selected from the LANL database using BLAST (allowing up to 10 hits per sequence; expect value = 1 × 10^−30^). The resulting 9922 sequences were aligned against the HBX2 subtype B reference strain and the alignment was stripped off the major resistance mutations listed by the IAS-USA [22]. A maximum likelihood phylogeny of the alignment was reconstructed in FastTree version 2.1.9 [23] assuming a GTR model of molecular evolution with gamma distributed rate heterogeneity. The resulting phylogeny was used to identify all sub-epidemics with at least two samples for which a minimum of 80 per cent of the samples were from Switzerland [14]. In the following we refer to these Swiss sub-epidemics as clusters. For computational reasons any cluster that contained more than 250 sequences was split at the root of the phylogeny and considered as two separate clusters.

For each of the major resistance mutations we selected all clusters that contained at least one sequence in which the respective mutation was present. From the remaining samples that did not have the respective mutation, we removed sequences in which any of the other major resistance mutations were present (≤ 10%). Hence, the sequences in the resulting clusters are either ‘truly sensitive’, or contain the respective resistance mutation. From the resulting resistance mutation related data sets (RMDS) we included only those that met the following two criteria: Summing over all clusters (i) the total number of sequences is ≥ 25 and (ii) the number of resistant sequences is ≥ 10 (see Table 1). Numbers of resistant and total samples per RMDS per cluster are illustrated in S1 Fig. More detailed cluster characteristics are given in Table 3 within S1 Text. Exemplary analysis files are provided in S1 File.

**Table 1:** Resistance mutations with numbers of corresponding clusters and samples, related drugs and drug usage dates within Switzerland.

	nRTI									NNRTI					PI
Resistance mutation	41L	67N	70R	184V	210W	215D	215S	215Y	219Q	103N	108I	138A	181C	190A	90M
Number (#) of clusters of size ≥ 2	56	23	19	35	18	18	16	25	20	25	10	46	8	8	14
# Sequences in clusters	927	667	712	1011	481	569	494	807	605	725	334	1014	329	311	389
# Resistant samples in clusters	93	39	26	44	26	41	31	28	28	38	11	109	10	12	38
Drug (SHCS drug codes)	AZT D4T	AZT D4T	AZT D4T	3TC ABC FTC	AZT D4T	AZT D4T	AZT D4T	AZT D4T	AZT D4T	NVP EFV	NVP EFV	RPV	NVP EFV ETV RPV	NVP EFV	NFV SQV
Drug usage ≥ 1%	1987	1987	1987	1995.5	1987	1987	1987	1987	1987	1997	1997	2013	1997	1997	1996
Drug usage < 1%	-	-	-	-	-	-	-	-	-	-	-	-	-	-	2008

nRTIs: Resistance mutation related to nucleoside/nucleotide reverse transcriptase inhibitors

NNRTIs: Resistance mutation related to non-nucleoside reverse-transcriptase inhibitors

PIs: Resistance mutation related to protease inhibitors

‘Drug usage ≥ 1%’ refers to the time at which the respective drug was prescribed to a minimum of one percent of patients within the SHCS. If multiple drugs are associated with a resistance mutation the earliest date is used. Accordingly, ‘Drug usage < 1%’ refers to the time when the respective drugs are no longer used in ≥ 1% of the patients.

### Phylodynamic analyses

The 15 RMDS were analysed using the multi-type birth–death model [18] in BEAST2 [24]. A single analysis was set up for each of the resistance mutations. The sequences were annotated with their date of sampling and type (sensitive or resistant) and the phylogenies for each cluster were reconstructed jointly with the epidemiological parameters. While the tree topology, tree height and length, the branch rate variation and sampled ancestors [25] were estimated separately for each of the clusters, the substitution rate and epidemiological parameters were estimated jointly for all clusters. The parameters that were estimated jointly for all clusters associated with a particular resistance mutation were informed by the phylogenies of these clusters together. For example, we estimate a single resistance evolution rate for each resistance mutation, such that all clusters contribute to the estimate of the resistance evolution rate. The unit of time used in our analyses is years, such that all rates estimated here are average rates per lineage per year.

Fig 1 depicts the two-type birth–death model employed. Transmission events can only occur within each type, i.e. a transmission event caused by a sensitive strain results in a new infection with a sensitive strain and likewise for resistant strains. The migration rates estimated from sensitive to resistant lineages and back are equivalent to population-level rates of resistance evolution and reversion, respectively. Specifically, moving from the sensitive compartment to the resistant compartment is modelled through a resistance evolution rate, the opposite direction is determined by the resistance reversion rate. Both rates are assumed to be zero before significant usage of the related drug(s) in Switzerland. We consider drug usage as significant whenever the respective drug is prescribed to a minimum of one percent of patients within the SHCS. The NNRTI-related mutation 138A is an exception because it occurred in 0.5% to 5% of viruses from treatment-naïve patients even before the introduction of the related drug rilpivirine [26, 27]. Hence, we do not assume the resistance evolution and reversion rates for the 138A mutation to be zero at any time. Instead we allow the rate to change in 2013, when usage of the respective drug in Switzerland became significant. The protease inhibitors related to resistance mutation 90M have been used in less than 1% of patients after 2008. Therefore, we allow the resistance evolution and reversion rates for the 90M mutation to change in a piecewise constant fashion, such that it is zero before 1996 and we obtain one estimate for the time during and one for after significant drug usage in Switzerland.

The effective reproduction number of the sensitive type through time, which is the quotient of the sensitive transmission rate over the removal rate: *R*_*s*_ = λ_*s*_/*δ*, is allowed to change over seven-year intervals (before 1994, 1994-2001, 2001-2008, 2008-2015) in a piecewise constant fashion. We estimate a constant between-host transmission ratio

$$\begin{array}{c} r_{\lambda }=\frac{\lambda_{r}}{\lambda_{s}} \end{array}$$

between the per lineage resistant transmission rate λ_*r*_ and the sensitive transmission rate λ_*s*_. Hence, *r*_λ_ < 1 implies that there is a fitness cost associated with a resistance mutation. On the other hand *r*_λ_ > 1 suggests that the resistance mutation confers an advantage to the viral strain. We assume a joint removal rate *δ* for both types.

The analyses were conducted using a HKY substitution model with gamma distributed rate heterogeneity and a relaxed clock model with lognormally distributed branch rates. The substitution rate was fixed to 2.55 ⋅ 10^−3^ [28]. For each cluster sampling was assumed to have started at the sampling time of its earliest sequence. We estimate a constant sampling proportion *p* with its prior distribution centered around 22%, which results from considering that about 50% of the HIV infected population in Switzerland is included in the SHCS, 67% of which have successfully been sequenced [21] and 65% of which are treatment naïve. Upon sampling, individuals are removed from the infectious pool at a removal probability *r*. This means that we allow sampled ancestors [25] in the reconstructed phylogenies implying that infected individuals may still transmit to others after they have been diagnosed. The prior distributions employed are listed in Table 2.

**Table 2:** Prior distributions for the birth–death model parameters.

	*R*_*s*_	*δ*	*r*_λ_	*p*	resistance evolution rate	resistance reversion rate	removal probability
	LogN(0,1.25)	LogN(-1,0.5)	LogN(0,0.5)	Beta(22,78)	Exp(1)	Exp(1)	Unif(0,1)

Multiple independent instances were run for each RMDS, which were then combined, resulting in a combined Markov chain length of at least 250 million after burn-in. The resulting effective sample size of each parameter estimated is greater or equal to 200. This unusually long chain length was necessary due to the setup of the analyses: In each analysis we reconstruct the phylogenies for 8 to 56 clusters, many of which are very small (down to 2 samples), but some of which are fairly large (up to 184 samples), which makes operator optimisation difficult.

### Ethics statement

The SHCS was approved by the ethics committees of the participating institutions (Kantonale Ethikkommission Bern, Ethikkommission des Kantons St. Gallen, Comite Departemental d’Ethique des Specialites Medicales et de Medicine Communataire et de Premier Recours, Kantonale Ethikkommission Zurich, Repubblica e Cantone Ticino—Comitato Ethico Cantonale, Commission Cantonale d’Étique de la Recherche sur l’Être Humain, Ethikkommission beider Basel; all approvals are available on http://www.shcs.ch/206-ethic-committee-approval-and-informed-consent), and written informed consent was obtained from all participants.

## Results

**Fig 2:**
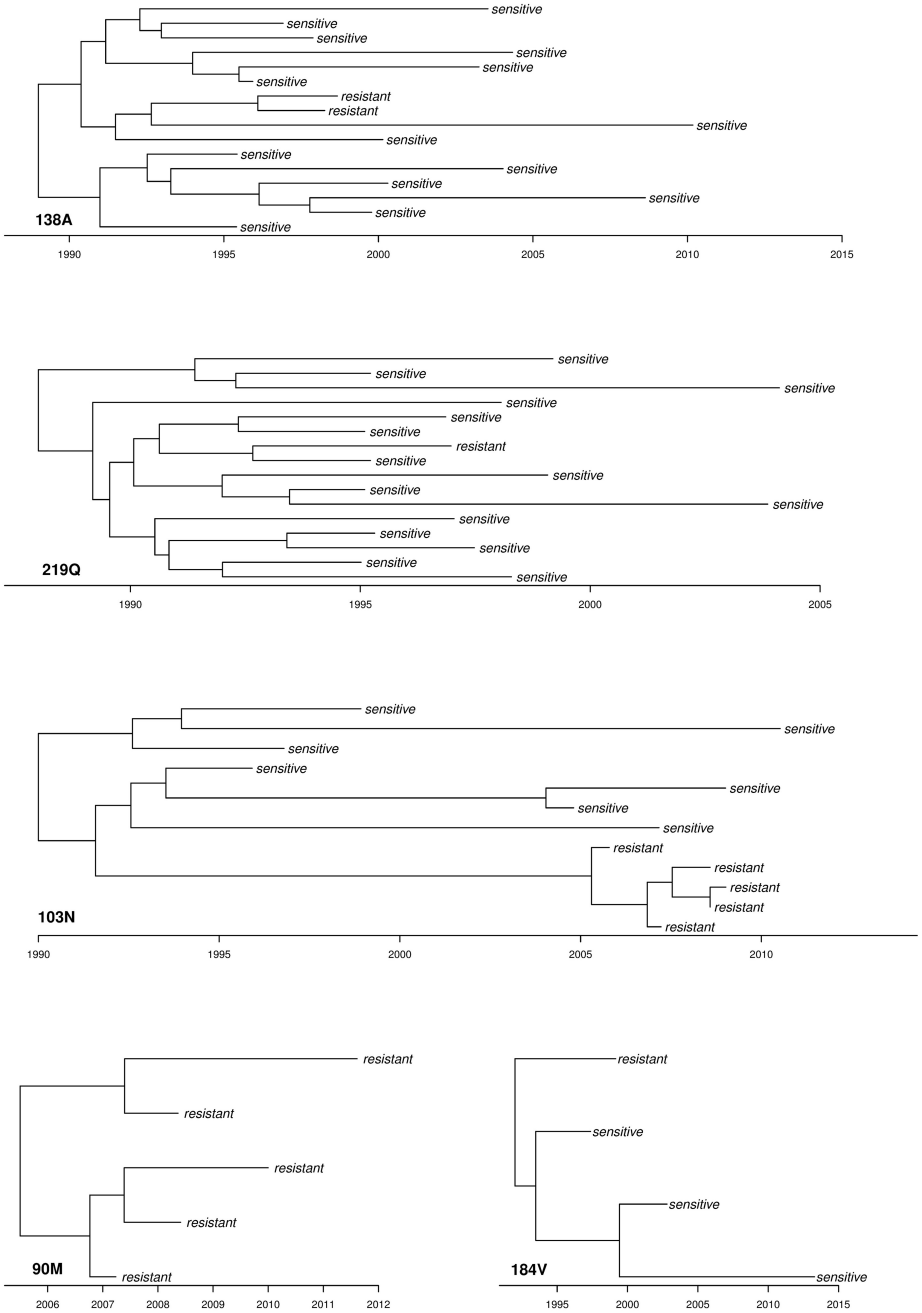
Maximum clade credibility trees of one cluster per drug class. Summary of the posterior distribution of the reconstructed phylogeny for one exemplary cluster in the 219Q, 138A, 184V, 103N and 90M RMDS. Exemplarily, the 103N cluster contains five resistant and seven sensitive samples. It has one sampled ancestor (indicated by the resistant sample with zero branch length), indicating that the respective patient transmitted to at least one other person after having been diagnosed with HIV.

After applying the selection criteria described in the Methods section, we obtained 15 RMDS with 8 (181C & 190A) to 56 (41L) clusters per resistance mutation comprising a total of 311 (190A) to 1014 (138A) sequences (Table 1). The total number of patients included in the study after applying the above criteria is 2614. Most clusters contain very few resistant samples. Sensitive samples are allowed to occur in multiple RMDS. Histograms of the numbers of (i) all and (ii) resistant samples per cluster are shown in S1 Fig. As an example of the resulting reconstructed cluster phylogenies, Fig 2 shows the maximum clade credibility trees of one cluster per drug class.

We were unable to obtain reliable estimates for the 215Y RMDS, as the MCMC did not converge even after more than 2.400 million combined MCMC steps (for details see Section 3.1 within S1 Text). Hence we present the results from the remaining 14 RMDS only.

**Fig 3:**
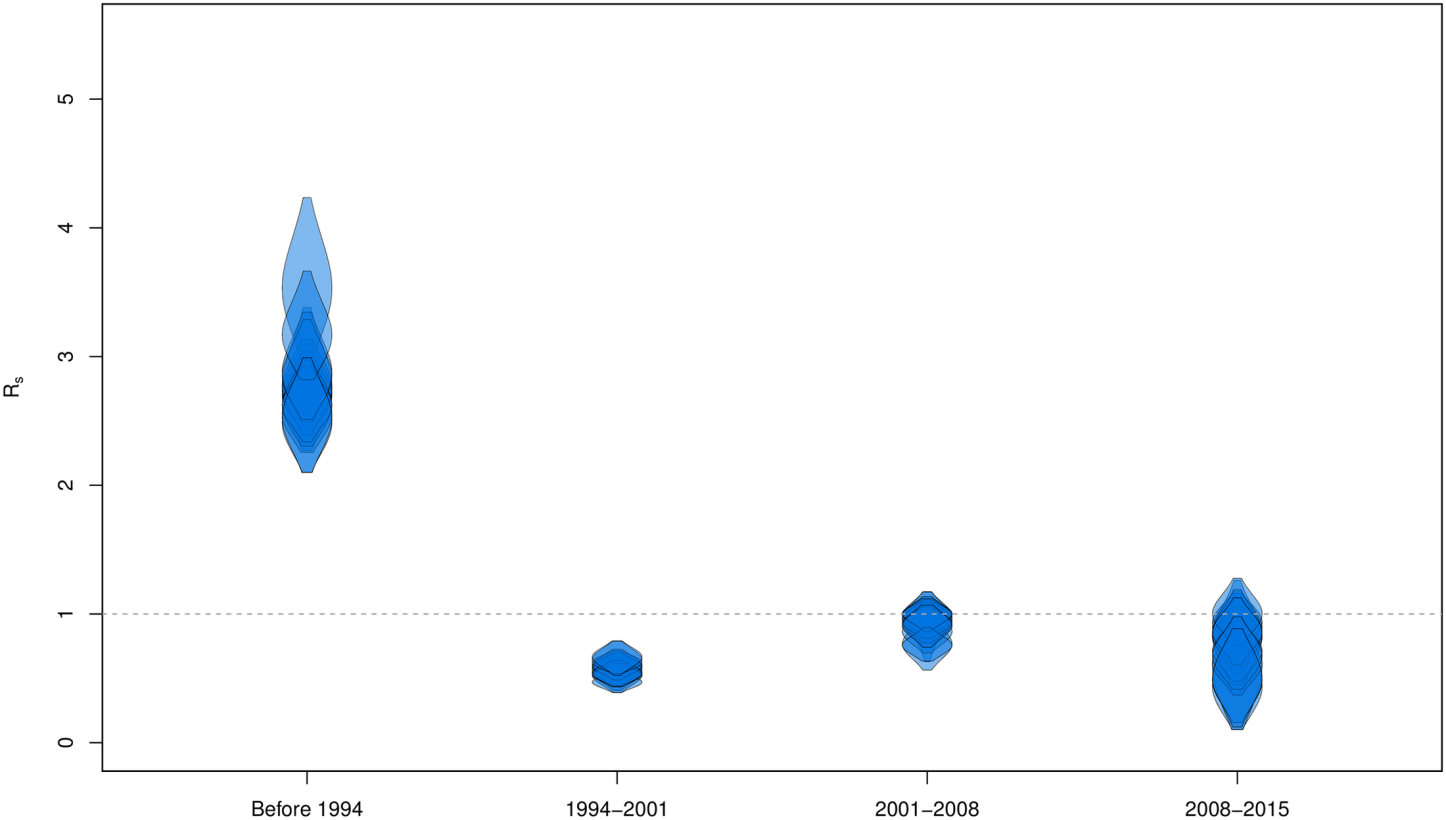
Estimates of the effective reproduction number *R*_*s*_ of the sensitive strains through time. Time has been partitioned into 4 fixed time intervals: before 1994, 1994-2001, 2001-2008, 2008-2015. For each time interval there are 14 estimates, one from each of the 14 resistance-mutation data sets (RMDS). The violin plots show the 95% HPDs of the *R*_*s*_ estimates.

We quantify the transmission dynamics of the drug sensitive virus samples by estimating the effective reproduction number, *R*_*s*_, through time. This yields one estimate for each of the 14 RMDS, which agree with one another (depicted by 14 overlapping violin plots per time interval, Fig 3). Our results suggest that *R*_*s*_ was above 2 before 1994, below the epidemic threshold of one between 1994 and 2001 and around 1 after 2001.

**Fig 4:**
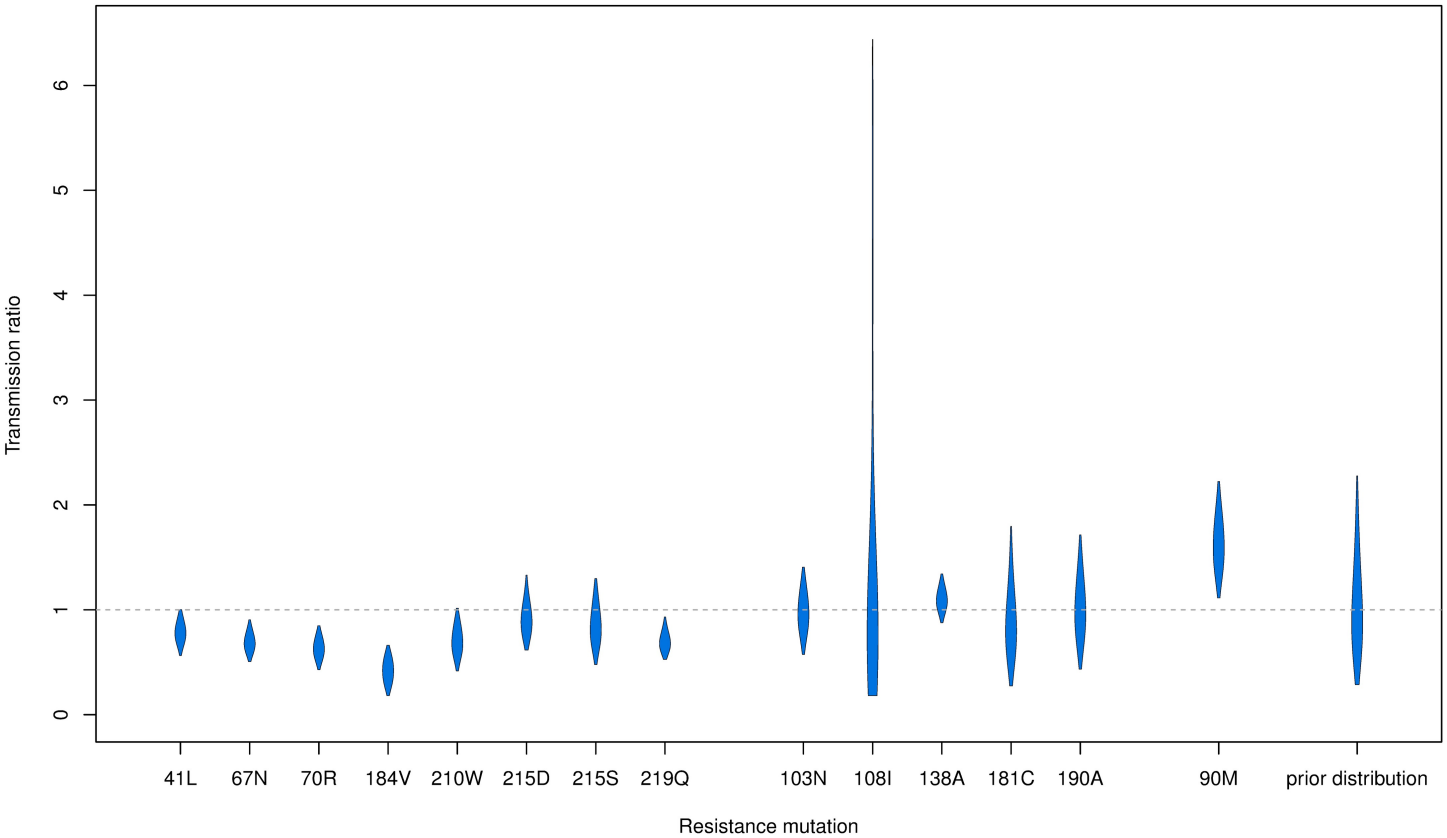
Estimates of the transmission ratio of the resistant strains during consumption in Switzerland. For each resistance mutation we estimate a between-host transmission ratio *r*_λ_ = λ_*r*_/λ_*s*_ between the per lineage resistant transmission rate λ_*r*_ and the sensitive transmission rate λ_*s*_. The respective drug consumption dates are given in Table 1. The violin plots show the 95% HPD intervals of the *r*_λ_ estimates. The same prior distribution was employed for all analyses (plotted on the far right).

For each resistance mutation we quantify the between-host fitness cost as the ratio *r*_λ_ of the transmission rate of the drug resistant strains divided by the transmission rate of the drug sensitive strain. Among the considered resistance mutations, only the 90M mutation in the protease gene was found to have significantly higher fitness than the drug sensitive strains (Fig 4). The following mutations associated with resistance to reverse transcriptase inhibitors were found to be less fit than the sensitive strains: 67N, 70R, 184V, 219Q. The highest posterior density intervals of the transmission ratios for the remaining resistance mutations (41L, 103N, 108I, 138A, 181C, 190A, 210W, 215D, 215S) all included the threshold one, suggesting that these mutations do not have a significant effect on viral transmissibility within the Swiss HIV Cohort Study.

The estimated rates of resistance evolution and reversion are shown in S2 and S3 Figs, respectively. The median rates of resistance evolution during significant drug usage in Switzerland are between 0.005–0.13. The corresponding median rates of resistance reversion range from 0.03–0.20. In detail, the posterior estimates of the epidemiological parameter estimates are given in Table 4 within S1 Text.

Our results suggest that once the patient samples are taken, the patients are unlikely to continue transmitting to others. In fact, the median number of sampled ancestors for most RMDS clusters is zero. Very few clusters belonging to the 215D, 108I, 181C and 190A data sets have significantly more than zero sampled ancestors. Overall, the probability of being removed from the infectious pool upon sampling is above 89% (smallest median estimate) for patients infected with sensitive strains. There is much uncertainty in the estimates of the removal probability for resistant strains, which range from 44% (215D; 95% HPD: 0.7-84%) to 95% (138A; 95% HPD: 81-100%),

## Discussion

In this study we present a computational method to quantify the transmission cost of HIV-1 drug resistance mutations and assess it in the context of the Swiss HIV Cohort Study, as the transmission of drug resistant strains is a big threat to the success of global HIV-containment programs (e.g. test-and-treat) [29]. The base data set used here is intentionally limited to samples from patients that have not undergone antiretroviral therapy (ART) at the time of sampling. Including ART experienced individuals would be problematic, because the transmission rates estimated from ART-naïve vs. ART-experienced individuals are likely confounded by several factors such as behavioural differences due to awareness of HIV status and the effect of ART on viral load and persistence of drug resistance mutations [30–36].

Furthermore, we exclude resistance mutations that occur in very few samples, to avoid identifiability issues in our analyses. Our study includes eight major resistance mutations related to nucleoside reverse transcriptase inhibitors, five related to non-nucleoside reverse transcriptase inhibitors and one related to protease inhibitors.

Our selection criteria serve as a filter for those drug resistance mutations that are moderately prevalent and transmissible in Switzerland. Hence, they represent the most important drug resistance mutations to be considered in Switzerland from a clinical and public health perspective. However, this does not include very recently introduced drugs, as they may not cross our thresholds yet. More specifically, it is unlikely to have samples from ten or more patients with a specific mutation conferring resistance to a drug that was introduced less than two or three years ago. For our data set this would translate to any introductions after 2013.

Our approach is based on the assumption that a viral strain can be annotated with either of two types, sensitive or resistant. This is a strong simplification since the presence of multiple drug resistance mutations may impact viral fitness in a different way than a single mutation. However, most transmitted virus strains in this study contain either zero or one major resistance mutation. We did not include compensatory mutations in our analyses due to their rare occurrence in the data set. Furthermore, we do not distinguish among different risk groups in this study, as there does not appear to be any significant difference in TDR prevalence according to risk groups [37].

We are assuming that the transmission tree coincides with the phylogeny, which is based on assuming that there is little or no superinfection and that within-host coalescence time is short compared to the viral transmission time. In addition, we assume a constant between-host transmission ratio *r*_λ_ = λ_*r*_/λ_*s*_, which implies that transmission rate changes due to a changing number of susceptible individuals or a change in public health interventions have the same effect on sensitive and resistant hosts. This effect cancels out in the ratio. Note that between-host transmission fitness and within-host replication fitness are intertwined. Within-host replication fitness is one of the factors that contributes to the overall epidemiological fitness of a viral strain, which is commonly measured by the effective reproduction number [38].

There are two technical assumptions of the phylodynamic model that were made for computational reasons only: (1) the removal rate is constant through time and (2) resistance evolution rates can be averaged over (i) connected time periods of no or very little drug usage (< 1%) and (ii) connected time periods of relevant usage (> 1%), see Fig 1 within S1 Text (scenario A). For three of the RMDS (184V, 103N and 90M) we have performed the same analysis again with these assumptions relaxed to have changes in removal rates at two time points and direct dependance of the resistance evolution rates on the annual drug usage data in the SHCS (scenario B). While we see a small decrease in the estimated transmission ratios, the 95% HPDs largely overlap, resulting in qualitatively equivalent interpretations (see Section 1 within S1 Text, esp. Fig 3 within S1 Text). Additionally we have conducted a simulation study in which we simulate three sets of RMDS representative of the actual RMDS 184V, 103N and 90M under the more complex scenario B and reconstruct the trees and epidemiological parameters under the simpler scenario A. Although this does introduce some bias into our estimates we can robustly estimate the transmission ratio in all three simulation sets (see Section 2 within S1 Text, esp. Fig 5 within S1 Text). In particular, the simulation study presented in S1 Text shows that we can differentiate among different types of between-host transmission fitness despite differences in population sizes.

While there may be variation among individuals that confounds the potential effects of drug resistance mutations on transmission fitness, the overall patterns of viral fitness identified in this study are consistent with other measures of the fitness cost of resistance mutations such as site-directed mutagenesis or reversion rates [10–12]. Furthermore, Wertheim et al. [39] have recently applied a network approach to a large data set from the United States, obtaining results consistent with those presented here.

The 90M mutation in the protease and the 138A mutation in the reverse transcriptase have both previously been associated with TDR even in the absence of drugs [40, 41]. In this study, they are the only resistance mutations that have as many as ten resistant samples per cluster. In fact, the 138A mutation occurs in the SHCS as early as 1995 although the drug (rilpivirine) was only introduced in Switzerland in 2013. It appears to be a natural polymorphism, which is present in 0.5% to 5% of viruses from treatment-naïve patients, although it is more common in subtype C than B [26, 27, 40]. Before 2013 the median resistance evolution and reversion rates are 0.008 (95% HPD: [0.005-0.01]) and 0.04 (95% HPD: [0.008-0.08]), respectively. There is much uncertainty in the resistance evolution rate (median 0.14, 95% HPD: [0.00009-0.43]) and reversion rate (median 0.20, 95% HPD: [0.0004-0.82]) after 2013, which is due to the interval 2013–2015 being relatively short. We estimate a transmission ratio of 1.09 (median; 95% HPD: [0.88, 1.34]) confirming that its fitness is similar to that of sensitive strains.

The 90M mutation in the protease is another case of treatment-independent transmission [42, 43]. Our results suggest that it confers a significant fitness advantage over the sensitive lineages. While prescription of the drugs (saquinavir and nelfinavir) against which 90M confers resistance has ceased in Switzerland around 2008, the mutation still occurs in later samples. The latest sample showing the 90M mutation within a cluster was obtained in 2011. There are four 90M clusters that contain more than one resistant sample, three of which are driven by men having sex with men (MSM) and one that contains five resistant samples all of which are from heterosexual individuals falling together in a five-sample sub-cluster. Since this appears to reflect the situation in the Swiss HIV epidemic well [44], there does not appear to be a confounding effect due to risk group.

Note that these results are based on a relatively small sample size (Table 3 within S1 Text). However, others have also estimated a fitness advantage for the 90M mutation based on an independent data set [39]. Furthermore, it has been shown previously that 90M prevalence increased in the Swiss HIV cohort in recent years, although it’s occurrence in patients failing treatment is declining [6]. These studies thus support that the 90M mutation has either a fitness advantage or at least no significant fitness cost.

In contrast, the 184V mutation in the reverse transcriptase stands out as the one with the highest transmission cost (median transmission ratio: 0.43, 95% HPD: [0.18-0.66]). Being a major nRTI mutation the 184V is the only resistance mutation in this study that has no cluster with more than two resistant samples (apart from the 215Y RMDS for which we were not able to obtain reliable results, see Section 3.1 within S1 Text). Hence, it appears that while the 184V mutation evolves frequently under failing treatment, its high between-host transmission cost results in very short transmission chains.

For the 103N mutation in the reverse transcriptase we estimate a transmission ratio of 0.97 (95% HPD, [0.57-1.41]), which may imply that the mutation confers no significant fitness advantage or disadvantage. This major NNRTI mutation is associated with failure of current first-line treatment [2, 9, 45, 46] and currently is the most important NNRTI [47]. The lack of a disadvantage in transmission underlines the clinical importance of the 103N mutation in the context of TDR, particularly in resource limited settings.

For the remaining NNRTI mutations (108I, 181C and 190A) we obtain results very similar to the 103N results, although with larger 95% HPD intervals, particularly for the 108I RMDS, due to small sample sizes (10-12 resistant samples per RMDS).

Extensive propagation of the 103N and 90M mutations is unlikely in Switzerland and other resource-rich countries, where drug resistance testing is performed routinely to identify active drugs. Nevertheless, even in these settings it is crucial to diagnose HIV-positive individuals early to decrease the prevalence of transmitted drug resistance. The transmission potential of these mutations must be taken into account in the management of low-resource HIV-epidemics. Although the prevalence of transmitted drug resistance appears to be lower in African countries, for example, this may be due to the later introduction and roll-out of ART. Hence, there is a large risk of NNRTI-resistant viruses spreading quickly in such settings, which would make the management of the HIV-epidemics in resource-limited settings very difficult.

Our results regarding sampled ancestors and the removal probability suggest that patients infected with sensitive strains may be less likely to transmit after diagnosis than patients infected with resistant strains. However, there is considerable uncertainty in the removal probability estimates for resistant strains. This may be overcome by including all type B samples of the cohort (rather than only the treatment naïve first samples), which will be the subject of future work. In data sets where transmission frequently occurs after diagnosis and treatment, it would be advisable to include ART status into the analysis.

The merits of molecular epidemiological approaches have been highlighted in previous studies, particularly for cases in which genetic data is combined with epidemiological, demographic and clinical data [19, 41, 48–52]. However, this is one of the first studies presenting a phylogenetic approach that allows direct quantification of population-level fitness costs of HIV resistance mutations from viral sequence data (annotated with sampling date and resistance type) alone. Hence, this approach may become particularly useful in assessing the risk of TDR in resource limited settings where resistance testing is possible but epidemiological, demographic and clinical data are missing. Furthermore, our approach is not only applicable to HIV but to any measurably evolving pathogen.

## Supporting information

S1 Fig Histograms of the number of (i) all and (ii) resistant samples per cluster per resistance mutation. (TIF)

S2 Fig Estimates of the resistance evolution rates during drug consumption in Switzerland. The violin plots show the 95% HPD intervals of the resistance evolution rate estimates for each resistance mutation. An exponential prior distribution with mean 1 was employed for all analyses.
(TIF)

S3 Fig Estimates of the resistance reversion rates during drug consumption in Switzerland. The violin plots show the 95% HPD intervals of the resistance reversion rate estimates for each resistance mutation. An exponential prior distribution with mean 1 was employed for all analyses.
(TIF)

S1 Text SHCS re-analysis and simulation study. Description and results of reanalysis under complex model, simulation study and supplementary information on clusters and posterior rate estimates.
(PDF)

S1 File XML files. Compressed data file containing analysis files for phylodynamic HIV data analysis under scenarios A and B as well as the XML files for the simulation and reanalysis of RMDS. The analyses were run with BEAST version 2.4.6 and require the bdmm package version 0.2 and its dependencies.
(ZIP)
