# Supplementary figures and images for: Quantifying the fitness cost of HIV-1 drug resistance mutations through phylodynamics
Source: PLoS Pathog. 2018 Feb 20;14(2):e1006895. doi: 10.1371/journal.ppat.1006895 (PMC5877888; doi:10.1371/journal.ppat.1006895)

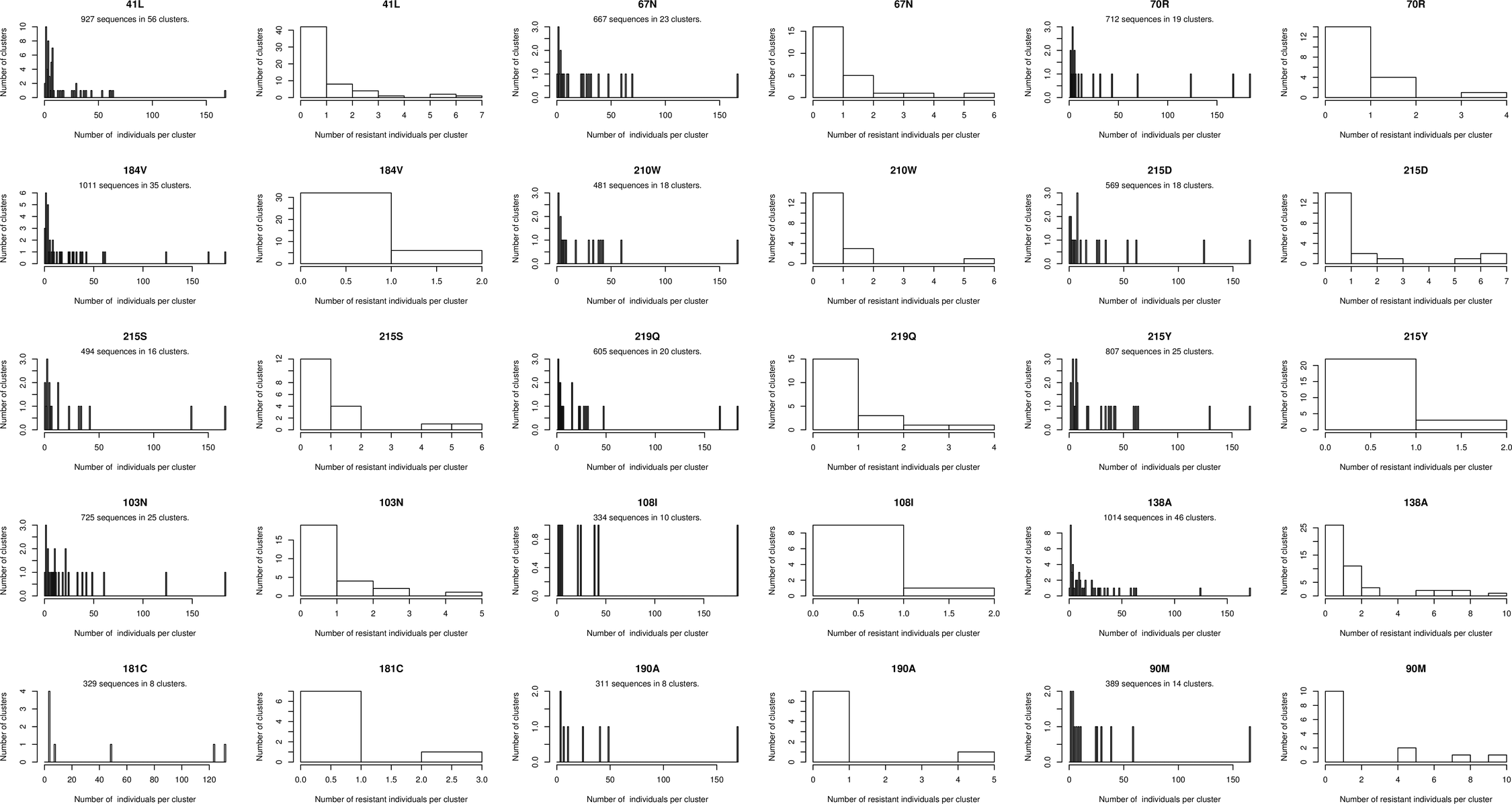

Supplement: S1 Fig — (TIF) [file ppat.1006895.s001.tif]

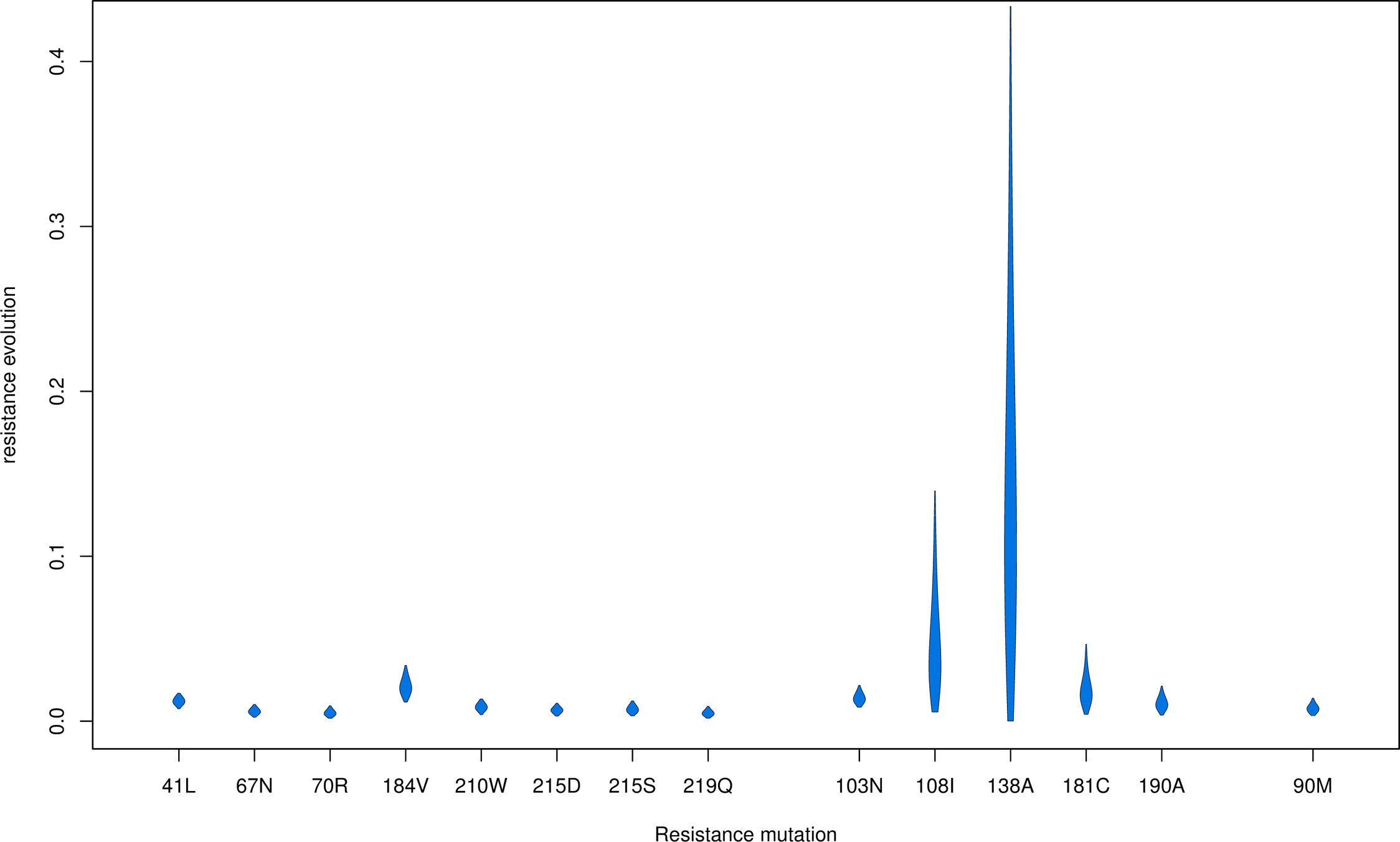

Supplement: S2 Fig — The violin plots show the 95% HPD intervals of the resistance evolution rate estimates for each resistance mutation. An exponential prior distribution with mean 1 was employed for all analyses. (TIF) [file ppat.1006895.s002.tif]

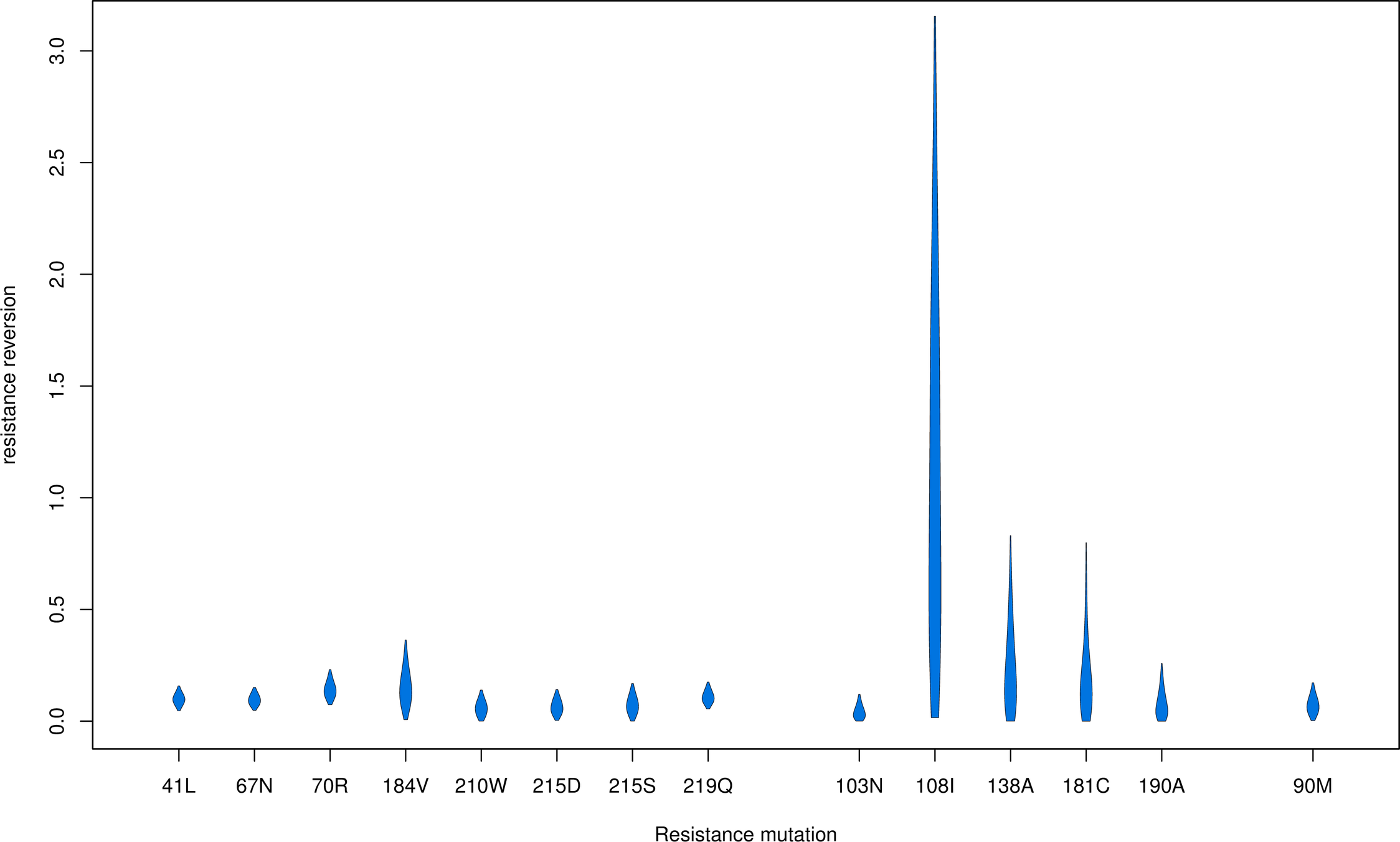

Supplement: S3 Fig — The violin plots show the 95% HPD intervals of the resistance reversion rate estimates for each resistance mutation. An exponential prior distribution with mean 1 was employed for all analyses. (TIF) [file ppat.1006895.s003.tif]
