# Supplementary material for: Quantifying the fitness cost of HIV-1 drug resistance mutations through phylodynamics
Source: PLoS Pathog. 2018 Feb 20;14(2):e1006895. doi: 10.1371/journal.ppat.1006895 (PMC5877888; doi:10.1371/journal.ppat.1006895)
Supplement: S1 Text — Description and results of reanalysis under complex model, simulation study and supplementary information on clusters and posterior rate estimates. (PDF) [file ppat.1006895.s004.pdf]

Supplementary Text S1

1 Reanalysis of SHCS data including drug usage data and changes in recovery rate

The phylodynamic analyses described in the main text were repeated under a more complex model specification for three of the RMDS: 184V, 103N and 90M. There are two differences in the model specification, see Figure 1 B:

1. Since we know how drug usage changed over time in percentage treated in the Swiss HIV cohort study (SHCS), we have implemented a variation of our model that estimates a resistance evolution rate which is proportional to the drug usage percentage of the relevant drug(s). Hence, we estimate a scaling factor instead of the resistance evolution rate itself. Tables 1-2 contain the relevant drug usage percentages in the SHCS per year.

2. We estimate the removal rate δ as a piecewise constant rate, with rate changes allowed at two time points: (i) in 2000 to account for the introduction of boosted protease inhibitors and (ii) in 2008 to account for the introduction of integrase inhibitors, since both events have likely caused a reduction in the time until infected individuals become virally suppressed and are hence removed from the infectious pool. The resulting effective reproduction numbers in the susceptible population and the transmission ratios r_λ of the respective resistance mutations are summarized in Figures 2-3.

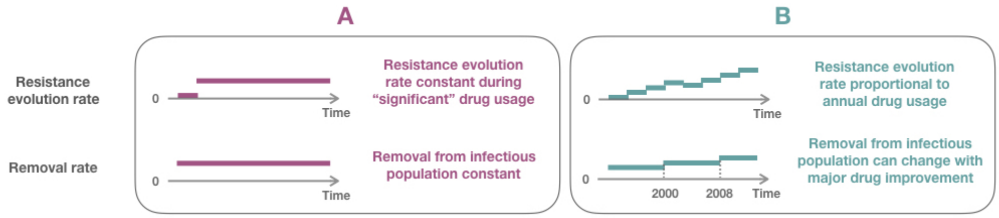

Fig 1. Schematic presentation of the simulation and analysis setups. The results presented in the main text where obtained using analysis setup A. In the simulation study scenario B was assumed during simulation, inference was done under scenario A. The SHCS results shown below were inferred with analysis setup B.

Table 1. Percentage of SHCS patients treated with one of the drugs associated with the 90M, 103N and 184V mutations, respectively, per year from 1993 to 2003.

	1993	1994	1995	1996	1997	1998	1999	2000	2001	2002	2003	mutation
NFV or SQV	0	0.0002	0.0012	0.0124	0.2211	0.4006	0.4172	0.3208	0.2684	0.2097	0.1602	90M
EFV or NVP	0	0	0	0.0002	0.0131	0.0514	0.2169	0.2886	0.3151	0.3257	0.3668	103N
3CT or ETC	0.0007	0.0016	0.0265	0.4520	0.6257	0.5923	0.6056	0.6030	0.6078	0.5948	0.6016	184V

The analysis setup was identical to the analyses described in the main text. In particular, the same prior distributions were employed

Table 2. Percentage of SHCS patients treated with one of the drugs associated with the 90M, 103N and 184V mutations, respectively, per year from 2004 to 2014.

	2004	2005	2006	2007	2008	2009	2010	2011	2012	2013	2014	mutation
NFV or SQV	0.1141	0.0812	0.0536	0.0347	0.0024	0.0026	0.0021	0.0015	0.0010	0.0009	0.0003	90M
EFV or NVP	0.3748	0.3523	0.3593	0.3715	0.3844	0.3894	0.4005	0.4088	0.4075	0.3857	0.3484	103N
3CT or ETC	0.6148	0.6497	0.7005	0.8569	0.8156	0.8394	0.8677	0.8952	0.8967	0.9145	0.8921	184V

Fig 2. Estimates of the effective reproduction number R_s of the sensitive strains through time. Time has been partitioned into 4 fixed time intervals: before 1994, 1994-2001, 2001-2008, 2008-2015. For each time interval there are three estimates, one from each of the resistance-mutation data sets 184V, 103N and 90M. The violin plots show the 95% HPDs of the R_s estimates.

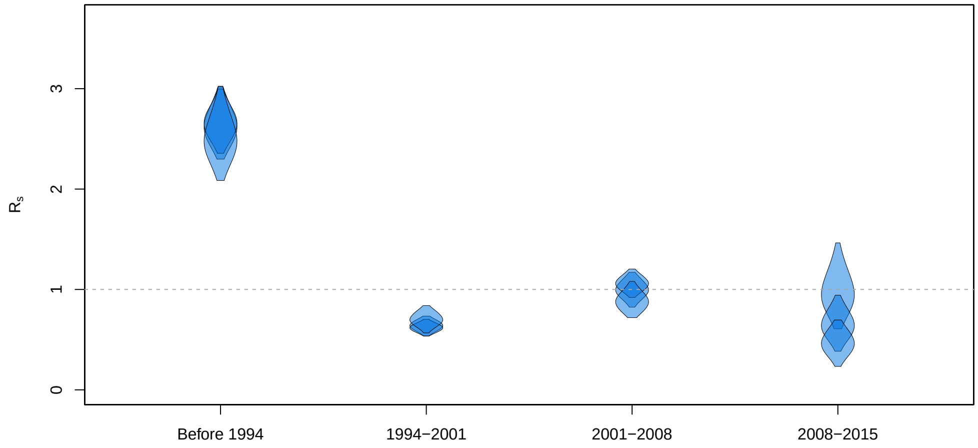

2 Simulation study: How well does the simple model capture the complex transmission dynamics?

In order to understand the effect of the simplifying assumptions that we made in the analyses under scenario A, we conducted a simulation study in which we simulated three sets of resistance mutation data sets (RMDS) under the complex model scenario B, which we then analysed assuming the simpler scenario A. The difference between the two scenarios is illustrated in Figure 1. The simulation parameters where chosen such that each of the three sets represents one of the three resistance mutations 184V, 103N and 90M. That is, in the 184V-like set of simulations we assume that there is a between-host fitness cost, for the 103N-like set we assume near-neutrality and for the 90M-like set we assume a fitness advantage for the strains carrying the mutation.

2.1 Simulating resistance-mutation data sets

For each of the three sets we simulated 40 replicates under scenario B. For each replicate a transmission tree was simulated using MASTER [?]. The simulated outbreaks start with a single host infected with a sensitive strain in year 1982. The effective reproduction number of the sensitive strains is piecewise constant, with

Fig 3. Estimates of the transmission ratio of the resistant strains during consumption in Switzerland. For each resistance mutation we estimate a between-host transmission ratio $r_\lambda = \lambda_r/\lambda_s$ between the per lineage resistant transmission rate λ_r and the sensitive transmission rate λ_s. The violin plots show the 95% HPD intervals of the r_λ estimates.

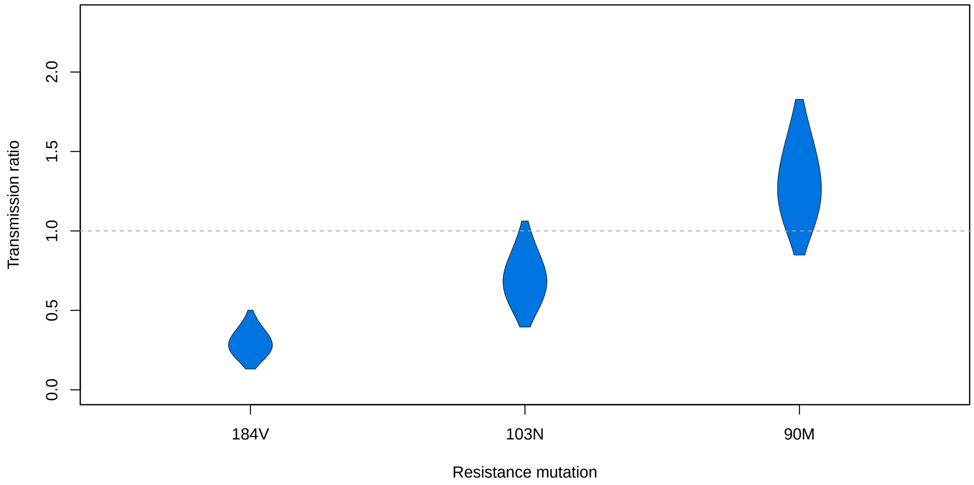

changes in years 2000, 2007 and 2014. The transmission ratio r_λ is set to 1.61 for the 90M-like simulation set, 0.96 for the 103N-like set and 0.41 for the 184V-like set. While the rate of resistance evolution changes annually, proportional to the respective drug usage in the SHCS (Tables 1-2), the resistance reversion rates are constant through time. The recovery rate increases twice, in 2000 and in 2008, with a population average infectious period of about 3.5, 3 and 2.5 years, assuming that infected individuals get diagnosed and successfully treated faster in the recent periods due to major improvements in antiretroviral therapy. The sensitive and resistant sampling proportions are set to zero until the time the first sensitive (or resistant) sample occurred in the respective SHCS RMDS and is a positive constant afterwards. An individual simulation was stopped when 150 samples (representing infected hosts) have been generated and was accepted as valid if it had a minimum of 1 resistant sample and a total (sum over sensitive and resistant samples) of at least 2 samples.

From the transmission trees generated with MASTER, sequence alignments of length 2000 were simulated using SeqGen [**?**]. The sequences were generated under the Hasegawa-Kishino-Yano (HKY) model of sequence evolution and an uncorrelated relaxed clock model assuming log-normally distributed rates of evolution among the branches of the tree with a mean substitution rate of 2.55 $\times 10^{-3}$ and a standard deviation of 0.5. The branch rate variation was included in order to mimic variation in the trees that may be caused by differences in within-host replicative dynamics.

For each replicate we simulated 10 sequence alignments representing 10 transmission chains of randomly varying sizes (from 2-150, see Figure 4).

2.2 Re-estimating simulated transmission fitness

The analysis setup for the simulated data sets was identical to the SHCS analyses described in the main text. In particular, the same prior distributions were employed,

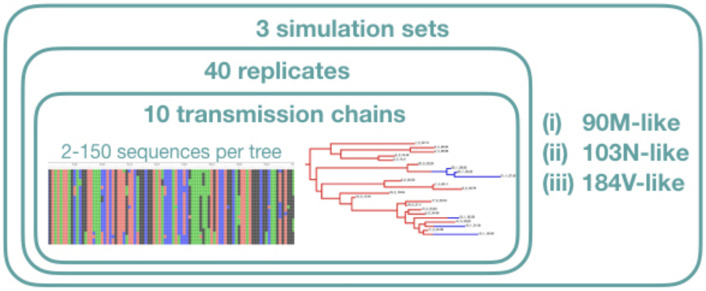

Fig 4. Overview of the simulation study.

with one exception: For the resistance evolution and reversion parameters a Exp(0.01) prior distribution was used in the simulation study. Although the resistance evolution and reversion rates were robust to different prior distributions in the SHCS analyses, the estimates of those parameters in the simulation study were not, instead they differed when different prior distributions were employed.

Analysing the RMDS under the simplified model scenario A leads to a reduction in the number of parameters to be estimated that reduces the computational complexity and improves identifiability of the epidemiological parameters. The price we usually pay for this is a loss of accuracy. The aim of this simulation study was to understand how well we can still estimate the between-host transmission fitness. The accuracy of the reconstructed transmission ratios r_λ is 87%, 73% and 100%, for the 90M-like, 103N-like and 184V-like RMDS, respectively, two of which are below the desired 95% accuracy. However, as depicted in Figure 5, accuracy is good and the simulation replicates for which r_λ is inaccurately estimated (i.e. the 95% highest posterior density interval does not contain the true value), the estimate is close to the truth and qualitatively correct. Indeed, all 184V-like simulation replicates yield a r_λ significantly smaller than 1, all 90M-like simulation replicates yield a r_λ significantly larger than 1, and all 103N-like simulation replicates yield a r_λ around or close to 1.

3 Supplementary information for the scenario A analyses presented in main text

Table 3 gives an overview of cluster the characteristics per RDMS.

mutation	nrSensitive	nrResistant	firstSen	lastSen	firstRes	lastRes
41L	0	2			2003.21	2007.15
	7	1	1995.53	2009.68	2006.34	2006.34
	6	2	1995.45	2003.16	1998.75	2006.32
	58	3	1993.1	2011.21	1998.52	2004.78
	3	1	1997.23	2008.29	2003.75	2003.75
	1	1	1996.37	1996.37	1995.88	1995.88
	7	1	1995.53	2001.95	1995.76	1995.76
	3	1	2008.62	2010.83	1998.38	1998.38
	27	1	1995.78	2013.8	2004.46	2004.46
	4	1	1991.97	2000.63	1997.28	1997.28
	2	6	1997.22	2003.39	2001.18	2012.96
	1	1	1999.24	1999.24	2003.87	2003.87
	6	3	1997.12	2006.91	1999.17	2001.95
	42	2	1995.57	2009.02	2001.68	2004.29
	165	3	1994.92	2014.23	1999.76	2009.98

mutation	nrSensitive	nrResistant	firstSen	lastSen	firstRes	lastRes
	7	1	1995.94	1999.67	1999.87	1999.87
	5	1	1992.83	2014.14	1996.25	1996.25
	1	1	1999.97	1999.97	2001.7	2001.7
	1	1	2000.89	2000.89	2000.03	2000.03
	29	1	1995.15	2011.74	2002.82	2002.82
	1	1	1998.96	1998.96	1997.7	1997.7
	33	1	1995.16	2012.77	1995.74	1995.74
	6	1	1997.11	2008.93	2000.81	2000.81
	7	1	1996.48	2011.27	1996.52	1996.52
	5	2	1995.63	2010.41	1997.39	2013.78
	47	7	1994.91	2013.45	1997.77	2001.12
	0	4			2006.83	2012.51
	1	2	2013.41	2013.41	2009.14	2009.2
	1	6	2007.22	2007.22	2008.19	2012.64
	12	1	1995.38	2014.26	2009.52	2009.52
	24	2	1995.88	2014.95	2000.8	2004.5
	7	1	1996.14	2008.77	2010.82	2010.82
	61	1	1996.77	2015.19	2010.86	2010.86
	0	1			2005.68	2005.68
	17	1	1995.97	2010.73	1999.55	1999.55
	3	1	1996.22	2003.38	2001.39	2001.39
	1	1	1997.44	1997.44	2001.78	2001.78
	3	1	1996.89	2007.79	1999.8	1999.8
	3	1	1997.82	2007.45	2001.31	2001.31
	0	2			2012.06	2012.11
	1	2	2011.7	2011.7	2012.14	2012.17
	36	1	1993.08	2013.45	1997.64	1997.64
	2	1	2002.02	2002.02	1997.96	1997.96
	1	1	2008.9	2008.9	1999.44	1999.44
	4	1	2004.86	2008.86	1999.47	1999.47
	38	1	1995.92	2012.76	1998.08	1998.08
	63	1	1995.07	2009.18	1997.1	1997.1
	16	1	1995.51	2005.65	1998.12	1998.12
	0	3			2012.52	2012.6
	3	1	1997.23	2013.15	1999.02	1999.02
	6	1	2000.28	2014.52	2000.7	2000.7
	1	1	2006.91	2006.91	1997.93	1997.93
	4	1	1995.93	2007.47	1996.16	1996.16
	14	1	1995.93	2010.69	2010.75	2010.75
	6	1	1995.9	2006.44	2003.19	2003.19
	0	1			2013.19	2013.19
	3	1	1997.58	2013.41	2006.63	2006.63
	29	1	1996.13	2012.94	2005.48	2005.48
67N	0	6			2006.63	2014.31
	3	1	1997.23	2008.29	2003.75	2003.75
	27	1	1995.78	2013.8	2004.46	2004.46
	47	1	1994.91	2013.45	1995.54	1995.54
	1	2	2007.22	2007.22	1995.59	1999.78
	1	1	1999.24	1999.24	2003.87	2003.87
	1	1	2003.73	2003.73	1995.61	1995.61
	29	1	1996.13	2012.94	1995.67	1995.67
	3	2	2002.9	2011.88	2007.84	2007.84
	165	2	1994.92	2014.23	1999.76	2000.69
	9	1	1996.23	2009.85	2003.2	2003.2
	58	2	1993.1	2011.21	1998.52	2004.63
	10	1	2001.03	2013.81	2000.07	2000.07

mutation	nrSensitive	nrResistant	firstSen	lastSen	firstRes	lastRes
	31	1	1996.35	2013.54	1996.51	1996.51
	69	1	1994.91	2012.12	1997.43	1997.43
	0	1			2008.39	2008.39
	1	1	1995.91	1995.91	1999.94	1999.94
	21	3	1995.56	2014.91	2008.36	2013.05
	6	1	2008.29	2009.62	1996.75	1996.75
	21	2	1996.87	2014.43	1996.34	2006.13
	38	1	1995.92	2012.76	1998.08	1998.08
	24	1	1995.88	2014.95	1999.19	1999.19
	63	1	1995.07	2009.18	1997.1	1997.1
	0	4			2012.53	2014.58
70R	0	4			2007.18	2009.94
	1	2	2007.22	2007.22	1995.59	1999.78
	1	1	1999.24	1999.24	2003.87	2003.87
	4	1	1997.41	2010.08	1997.29	1997.29
	9	1	1996.23	2009.85	2003.2	2003.2
	5	1	1996.17	2008.14	1995.92	1995.92
	165	2	1994.92	2014.23	2001.5	2007.43
	31	1	1996.35	2013.54	1996.51	1996.51
	42	2	1995.57	2009.02	1991.8	1996.61
	5	1	1995.93	2001.3	1996.76	1996.76
	183	1	1993.37	2014.33	1997.27	1997.27
	69	1	1994.91	2012.12	1997.43	1997.43
	3	1	1995.68	1996.72	1997.92	1997.92
	1	1	1995.91	1995.91	1999.94	1999.94
	6	1	2008.29	2009.62	1996.75	1996.75
	11	2	1997.6	2010.48	2001.11	2002.68
	24	1	1995.88	2014.95	1999.19	1999.19
	123	1	1993.04	2015.1	1997.58	1997.58
	3	1	1995.76	2008.87	1995.94	1995.94
184V	61	1	1994.89	2012.54	2006.94	2006.94
	0	2			2003.86	2007.36
	24	1	1996.98	2012.84	2007.39	2007.39
	3	1	1997.23	2008.29	2003.75	2003.75
	0	1			2003.21	2003.21
	27	2	1995.78	2013.8	2000.29	2004.46
	183	1	1993.37	2014.33	2003.84	2003.84
	1	1	1999.24	1999.24	2003.87	2003.87
	6	2	1995.9	2006.44	2003.19	2004.14
	1	1	2000.89	2000.89	2000.03	2000.03
	58	2	1993.1	2011.21	1998.52	2004.63
	165	2	1994.92	2014.23	1996.28	2011.3
	0	1			2008.39	2008.39
	8	1	1995.71	2010.83	1999.21	1999.21
	8	1	1995.65	2011.15	2004.61	2004.61
	5	1	1995.62	2007.74	2010.67	2010.67
	1	1	1999.05	1999.05	2010.97	2010.97
	11	2	1997.6	2010.48	2001.11	2002.68
	3	1	1997.43	2004.12	2000.7	2000.7
	17	1	1995.97	2010.73	1999.55	1999.55
	3	1	1996.22	2003.38	2001.39	2001.39
	42	1	1995.57	2009.02	2001.68	2001.68
	3	1	1996.89	2007.79	1999.8	1999.8
	4	1	1999.56	2009.1	2003.43	2003.43
	36	1	1993.08	2013.45	1997.64	1997.64
	32	1	1995.82	2012.84	1999.15	1999.15

mutation	nrSensitive	nrResistant	firstSen	lastSen	firstRes	lastRes
	1	1	2008.9	2008.9	1999.44	1999.44
	0	1			1999.85	1999.85
	38	1	1995.92	2012.76	1998.08	1998.08
	25	1	1996.47	2013.31	1999.95	1999.95
	123	1	1993.04	2015.1	1996.93	1996.93
	3	1	1997.23	2013.15	1999.02	1999.02
	16	1	1995.51	2005.65	1999.03	1999.03
	1	1	2007.22	2007.22	1999.78	1999.78
	15	1	1995.56	2004.65	2000.03	2000.03
	29	1	1995.64	2013.64	1998.27	1998.27
	9	1	1997.12	2009.7	1999.5	1999.5
	5	1	1995.63	2010.41	2013.78	2013.78
215D	8	3	1996.43	2013.49	2004.23	2007.02
	0	1			2007.15	2007.15
	27	1	1995.78	2013.8	2004.46	2004.46
	15	1	1995.56	2004.65	1998.43	1998.43
	2	6	1997.22	2003.39	2001.18	2012.96
	165	1	1994.92	2014.23	2001.5	2001.5
	5	1	1995.63	2010.41	1997.39	1997.39
	47	7	1994.91	2013.45	1997.77	2001.12
	1	7	2007.22	2007.22	2008.19	2012.64
	24	2	1995.88	2014.95	2000.8	2004.5
	61	1	1996.77	2015.19	2010.86	2010.86
	0	2			2012.06	2012.11
	2	1	2002.02	2002.02	1997.96	1997.96
	123	1	1993.04	2015.1	1998.02	1998.02
	1	1	1996.22	1996.22	2002.94	2002.94
	33	1	2005.75	2014.26	2000.67	2000.67
	7	1	1996.48	2011.27	1997.1	1997.1
	4	1	1995.93	2007.47	1996.16	1996.16
	0	1			2013.92	2013.92
	3	1	1997.58	2013.41	2006.63	2006.63
215S	6	1	1995.45	2003.16	2006.32	2006.32
	0	1			2001.06	2001.06
	40	2	1995.61	2007.47	2003.52	2008.05
	5	1	1996.4	2005.48	1996.81	1996.81
	0	2			2005.68	2008.3
	129	6	1989.67	2012.24	2001.26	2005.53
	0	5			2006.83	2012.51
	1	2	1997.44	1997.44	2001.78	2008.41
	31	1	1996.35	2013.54	2009.38	2009.38
	12	1	1995.38	2014.26	2009.52	2009.52
	4	1	1995.76	2003.93	2005.84	2005.84
	21	2	1996.87	2014.43	1996.34	2006.13
	12	1	1995.59	2010.23	1995.99	1995.99
	165	1	1994.92	2014.23	2006.51	2006.51
	2	1	1997.33	1999.45	1997.22	1997.22
	2	1	1994.64	2000.61	2002.72	2002.72
	0	1			2013.19	2013.19
	33	1	1995.16	2012.77	2003.5	2003.5
215Y	61	1	1994.89	2012.54	2006.94	2006.94
	7	1	1995.53	2001.95	1995.76	1995.76
	3	1	2008.62	2010.83	1998.38	1998.38
	4	1	1991.97	2000.63	1997.28	1997.28
	165	2	1994.92	2014.23	1999.76	2005.73
	5	1	1992.83	2014.14	1996.25	1996.25

mutation	nrSensitive	nrResistant	firstSen	lastSen	firstRes	lastRes
	58	2	1993.1	2011.21	1998.52	2004.63
	33	1	1995.16	2012.77	1995.74	1995.74
	129	1	1989.67	2012.24	1996.24	1996.24
	7	1	1996.48	2011.27	1996.52	1996.52
	40	2	1995.61	2007.47	1996.54	1999.11
	1	1	1999.05	1999.05	2010.97	2010.97
	6	1	2008.29	2009.62	1996.75	1996.75
	17	1	1995.97	2010.73	1999.55	1999.55
	3	1	1996.22	2003.38	2001.39	2001.39
	42	1	1995.57	2009.02	2001.68	2001.68
	6	1	1995.45	2003.16	1998.75	1998.75
	36	1	1993.08	2013.45	1997.64	1997.64
	1	1	2008.9	2008.9	1999.44	1999.44
	38	1	1995.92	2012.76	1998.08	1998.08
	63	1	1995.07	2009.18	1997.1	1997.1
	16	1	1995.51	2005.65	1998.12	1998.12
	3	1	1997.23	2013.15	1999.02	1999.02
	29	1	1995.64	2013.64	1996.94	1996.94
	6	1	1995.9	2006.44	2003.19	2003.19
219Q	3	1	1997.23	2008.29	2003.75	2003.75
	27	1	1995.78	2013.8	2004.46	2004.46
	47	1	1994.91	2013.45	1995.54	1995.54
	1	2	2007.22	2007.22	1995.59	1999.78
	15	1	1995.56	2004.65	1997.51	1997.51
	1	1	2003.73	2003.73	1995.61	1995.61
	29	1	1996.13	2012.94	1995.67	1995.67
	3	2	2002.9	2011.88	2007.84	2007.84
	31	1	1996.35	2013.54	1996.51	1996.51
	183	1	1993.37	2014.33	1997.27	1997.27
	1	1	2012.95	2012.95	1998.42	1998.42
	1	1	1995.91	1995.91	1999.94	1999.94
	165	1	1994.92	2014.23	2000.69	2000.69
	5	1	1995.62	2007.74	2010.04	2010.04
	21	3	1995.56	2014.91	2008.36	2013.05
	6	1	2008.29	2009.62	1996.75	1996.75
	21	2	1996.87	2014.43	1996.34	2006.13
	0	4			2012.53	2014.58
	2	1	2011.46	2011.75	2002.04	2002.04
	15	1	1995.81	2009.21	2001.39	2001.39
210W	7	2	1995.72	2010.33	2005.6	2007.03
	33	1	1995.16	2012.77	2006.78	2006.78
	40	1	1995.61	2007.47	2003.52	2003.52
	2	6	1997.22	2003.39	2001.18	2012.96
	165	2	1994.92	2014.23	1999.76	2005.73
	5	1	1992.83	2014.14	1996.25	1996.25
	58	2	1993.1	2011.21	1998.52	2004.63
	29	1	1995.15	2011.74	2002.82	2002.82
	1	1	2006.77	2006.77	1996.24	1996.24
	17	1	1995.97	2010.73	1999.55	1999.55
	3	1	1996.22	2003.38	2001.39	2001.39
	42	1	1995.57	2009.02	2001.68	2001.68
	1	1	2008.9	2008.9	1999.44	1999.44
	4	1	2004.86	2008.86	1999.47	1999.47
	38	1	1995.92	2012.76	1998.08	1998.08
	3	1	1997.23	2013.15	1999.02	1999.02
	1	1	1996.22	1996.22	2002.94	2002.94

mutation	nrSensitive	nrResistant	firstSen	lastSen	firstRes	lastRes
	6	1	1995.9	2006.44	2003.19	2003.19
103N	7	5	1995.72	2010.33	2005.6	2008.83
	10	1	1995.63	2008.5	2000.45	2000.45
	38	1	1995.47	2011.82	2007.38	2007.38
	3	1	2011.24	2014.06	2007.35	2007.35
	24	1	1996.98	2012.84	2007.39	2007.39
	183	1	1993.37	2014.33	2003.84	2003.84
	8	1	1996.18	2011.65	2007.83	2007.83
	6	2	1995.9	2006.44	2003.19	2004.14
	58	3	1993.1	2011.21	2007.83	2009.94
	21	1	1995.56	2014.91	2007.97	2007.97
	13	2	1996.93	2013.24	2008.37	2012.66
	17	2	1995.97	2010.73	2003.91	2006.41
	8	3	1995.65	2011.15	2009.16	2009.65
	8	2	1995.66	2013.51	2009.77	2013.24
	1	1	2004.48	2004.48	2010.5	2010.5
	48	1	1995.68	2010.71	2010.52	2010.52
	6	1	2003.05	2011.95	2004.96	2004.96
	1	1	1999.05	1999.05	2010.97	2010.97
	0	1			2011.2	2011.2
	42	1	1995.57	2009.02	2001.68	2001.68
	33	1	2005.75	2014.26	2005.09	2005.09
	1	1	2007.22	2007.22	2011.84	2011.84
	3	1	1999.73	1999.77	2006.3	2006.3
	4	1	1999.56	2009.1	2003.43	2003.43
	123	1	1993.04	2015.1	2003.18	2003.18
	21	1	1995.57	2010.67	2010.88	2010.88
108I	3	1	2011.24	2014.06	2007.35	2007.35
	24	1	1996.98	2012.84	2007.39	2007.39
	21	1	1995.56	2014.91	2007.97	2007.97
	183	2	1993.37	2014.33	2002.33	2002.67
	38	1	1995.47	2011.82	1998.68	1998.68
	5	1	1995.93	2001.3	2005.26	2005.26
	2	1	1998.31	2010.72	2011.38	2011.38
	42	1	1995.57	2009.02	2001.68	2001.68
	4	1	1999.56	2009.1	2003.43	2003.43
	1	1	2011.58	2011.58	1996.79	1996.79
138A	2	8	1998.31	2010.72	2006.52	2011.38
	25	3	1996.47	2013.31	1999.95	2006.92
	0	10			2001.39	2013.95
	7	3	1995.88	2003.62	2003.47	2006.99
	2	6	1997.33	1999.45	1996.22	2007.24
	2	1	1999.12	2010.18	1996.02	1996.02
	1	7	1997.77	1997.77	2004.79	2010.02
	23	2	1995.51	2008.45	2003.14	2005.38
	165	7	1994.92	2014.23	1995.34	2014.46
	21	8	1995.56	2014.91	2004.23	2011.49
	6	1	1997.11	2008.93	2004.18	2004.18
	0	2			2000.03	2001.86
	1	6	2000.08	2000.08	2008.02	2014.29
	1	2	1996.49	1996.49	2004.49	2006.49
	21	1	1996.87	2014.43	1996.05	1996.05
	123	2	1993.04	2015.1	1996.06	1996.34
	0	2			1997.77	2006.21
	1	1	1997.16	1997.16	1998.78	1998.78
	36	1	1993.08	2013.45	1997.08	1997.08

mutation	nrSensitive	nrResistant	firstSen	lastSen	firstRes	lastRes
	42	1	1995.57	2009.02	1996.61	1996.61
	3	2	1998.32	2003.22	1996.83	1997.67
	1	2	2011.35	2011.35	2005.32	2008.82
	3	1	2009.18	2010.47	2006.81	2006.81
	10	1	2001.03	2013.81	2008.85	2008.85
	20	3	2004.47	2013.58	2008.94	2009.04
	14	1	1995.64	2007.68	2004.86	2004.86
	12	1	1995.38	2014.26	2009.52	2009.52
	15	1	1995.56	2004.65	2004.26	2004.26
	1	1	2003.69	2003.69	2004.86	2004.86
	29	1	1995.64	2013.64	2004.98	2004.98
	33	1	1995.16	2012.77	2009.98	2009.98
	1	1	2004.48	2004.48	2010.5	2010.5
	0	2			2010.94	2011.59
	0	2			1996.43	2005.93
	3	1	1999.1	2009.31	2002.47	2002.47
	21	1	1995.57	2010.67	2002.18	2002.18
	1	1	2002.54	2002.54	2000.83	2000.83
	14	2	1995.93	2010.69	1998.78	1999.19
	3	1	1997.1	2003.04	2006.17	2006.17
	3	1	1999.73	1999.77	2006.3	2006.3
	58	1	1993.1	2011.21	1997.43	1997.43
	47	1	1994.91	2013.45	1998.88	1998.88
	61	1	1994.89	2012.54	1997.29	1997.29
	63	1	1995.07	2009.18	2008.6	2008.6
	10	1	1996.43	2008.42	2003.24	2003.24
	0	2			2013.14	2013.2
	0	1			2013.75	2013.75
181C	7	1	1995.53	2009.68	2006.34	2006.34
	3	1	2011.24	2014.06	2007.35	2007.35
	3	1	1994.82	2003.18	2003.66	2003.66
	3	1	1997.23	2008.29	2003.75	2003.75
	48	1	1995.68	2010.71	2007.22	2007.22
	129	3	1989.67	2012.24	2007.3	2010.92
	123	1	1993.04	2015.1	1998.02	1998.02
	3	1	1997.58	2013.41	2006.63	2006.63
190A	165	5	1994.92	2014.23	2006.51	2013.56
	40	1	1995.61	2007.47	2007.3	2007.3
	3	1	1994.82	2003.18	2003.66	2003.66
	48	1	1995.68	2010.71	2007.22	2007.22
	10	1	1995.95	2013.12	2008.86	2008.86
	3	1	1999.73	1999.77	2006.3	2006.3
	24	1	1995.88	2014.95	1996.16	1996.16
	6	1	1995.9	2006.44	2003.19	2003.19
90M	21	5	1994.97	2011.15	2003.77	2006.64
	0	5			2007.18	2011.56
	3	1	1997.23	2008.29	2003.75	2003.75
	3	1	2008.62	2010.83	1998.38	1998.38
	1	1	1999.24	1999.24	2003.87	2003.87
	1	10	2009.81	2009.81	2005.37	2011.12
	58	1	1993.1	2011.21	2004.63	2004.63
	29	1	1995.15	2011.74	2002.82	2002.82
	6	1	1997.11	2008.93	2000.81	2000.81
	1	8	2010.56	2010.56	2008.95	2011.22
	38	1	1995.92	2012.76	1998.08	1998.08
	24	1	1995.88	2014.95	1999.19	1999.19

mutation	nrSensitive	nrResistant	firstSen	lastSen	firstRes	lastRes
	1	1	2007.22	2007.22	1999.78	1999.78
	165	1	1994.92	2014.23	2007.43	2007.43

Table 3. Overview of cluster characteristics per RDMS. Each line corresponds to one cluster, and the header abbreviations refer to the following: mutation - which RMDS does the cluster belong to; nrSensitive - the number of sensitive samples in the cluster; nrResistant - the number of resistant samples in the cluster; firstSen - the time when the first (i.e. oldest) sensitive sequence was sampled ; lastSen - the time when the last (i.e. most recent) sensitive sequence was sampled; firstRes- the time when the first (i.e. oldest) resistant sequence was sampled; lastRes - the time when the last (i.e. most recent) resistant sequence was sampled

Table 4 lists the posterior estimates of the effective reproduction number of the sensitive strains, the transmission ratio and the resistance evolution and reversion rates estimated under scenario A.

mutation	parameter	median	95% HPD
103N			
	$R_{s,\text{before1994}}$	2.7418	(2.4045-3.1105)
	$R_{s,1994-2001}$	0.5945	(0.5011-0.6968)
	$R_{s,2001-2008}$	0.9082	(0.7752-1.0274)
	$R_{s,2008-2015}$	0.8969	(0.6164-1.1809)
	transmission ratio r_λ	0.9581	(0.5592-1.3651)
	resistance evolution rate	0.0135	(0.0077-0.0202)
	resistance reversion rate	0.0304	(0-0.0937)
108I			
	$R_{s,\text{before1994}}$	2.7997	(2.285-3.401)
	$R_{s,1994-2001}$	0.6462	(0.4968-0.7912)
	$R_{s,2001-2008}$	0.9477	(0.6266-1.1682)
	$R_{s,2008-2015}$	0.4519	(0.1064-0.8209)
	transmission ratio r_λ	0.791	(0.1787-6.4336)
	resistance evolution rate	0.0425	(0.0056-0.1396)
	resistance reversion rate	1.1013	(0.0156-3.1517)
181C			
	$R_{s,\text{before1994}}$	3.5568	(2.9421-4.2367)
	$R_{s,1994-2001}$	0.525	(0.3895-0.6602)
	$R_{s,2001-2008}$	0.771	(0.5647-0.9839)
	$R_{s,2008-2015}$	0.6226	(0.0979-1.1613)
	transmission ratio r_λ	0.8659	(0.2634-1.7771)
	resistance evolution rate	0.0179	(0.0044-0.0466)
	resistance reversion rate	0.1504	(0-0.7867)
190A			
	$R_{s,\text{before1994}}$	2.5024	(2.0983-2.9422)
	$R_{s,1994-2001}$	0.5444	(0.4097-0.6809)
	$R_{s,2001-2008}$	0.9882	(0.81-1.174)
	$R_{s,2008-2015}$	0.4983	(0.1574-0.8585)
	transmission ratio r_λ	1.0102	(0.4594-1.7366)
	resistance evolution rate	0.0108	(0.0036-0.0213)
	resistance reversion rate	0.0682	(0-0.2586)
138A			
	$R_{s,\text{before1994}}$	2.7003	(2.4217-2.9803)
	$R_{s,1994-2001}$	0.4723	(0.3932-0.553)
	$R_{s,2001-2008}$	1.0012	(0.8925-1.1027)
	$R_{s,2008-2015}$	0.9414	(0.724-1.1748)
	transmission ratio r_λ	1.0903	(0.8849-1.3293)
	resistance evolution rate (before drug usage)	0.008	(0.0054-0.0109)

mutation	parameter	median	95% HPD
	resistance evolution rate	0.0463	(2e-04-0.1402)
	resistance reversion rate (before drug usage)	0.0379	(0.0084-0.0721)
	resistance reversion rate	0.0553	(0-0.2254)
184V			
	$R_{s,\text{before1994}}$	2.7151	(2.42-3.0277)
	$R_{s,1994-2001}$	0.5807	(0.5033-0.6693)
	$R_{s,2001-2008}$	1.0009	(0.8848-1.1085)
	$R_{s,2008-2015}$	0.6199	(0.3932-0.8707)
	transmission ratio r_λ	0.4101	(0.1981-0.6868)
	resistance evolution rate	0.0184	(0.0114-0.028)
	resistance reversion rate	0.0929	(7e-04-0.2196)
210W			
	$R_{s,\text{before1994}}$	2.5632	(2.1858-2.9289)
	$R_{s,1994-2001}$	0.5631	(0.4532-0.6848)
	$R_{s,2001-2008}$	0.8596	(0.6987-1.018)
	$R_{s,2008-2015}$	0.8296	(0.4114-1.2195)
	transmission ratio r_λ	0.6909	(0.4183-1.024)
	resistance evolution rate	0.0086	(0.0042-0.0134)
	resistance reversion rate	0.0472	(0-0.1163)
215D			
	$R_{s,\text{before1994}}$	2.7622	(2.3947-3.1683)
	$R_{s,1994-2001}$	0.5336	(0.4287-0.64)
	$R_{s,2001-2008}$	0.9684	(0.835-1.1116)
	$R_{s,2008-2015}$	0.8323	(0.5939-1.0529)
	transmission ratio r_λ	0.9019	(0.6016-1.2766)
	resistance evolution rate	0.0065	(0.0029-0.0105)
	resistance reversion rate	0.0534	(0.0036-0.1261)
215S			
	$R_{s,\text{before1994}}$	2.8829	(2.4775-3.3406)
	$R_{s,1994-2001}$	0.6086	(0.4863-0.7279)
	$R_{s,2001-2008}$	0.9317	(0.7827-1.0917)
	$R_{s,2008-2015}$	0.8535	(0.5275-1.1692)
	transmission ratio r_λ	0.8336	(0.4841-1.2828)
	resistance evolution rate	0.0071	(0.0036-0.012)
	resistance reversion rate	0.0561	(0-0.1349)
219Q			
	$R_{s,\text{before1994}}$	2.6481	(2.3144-3.0027)
	$R_{s,1994-2001}$	0.5924	(0.4872-0.6949)
	$R_{s,2001-2008}$	1.0065	(0.8786-1.1449)
	$R_{s,2008-2015}$	0.7207	(0.472-0.9785)
	transmission ratio r_λ	0.6793	(0.5017-0.8951)
	resistance evolution rate	0.0047	(0.0017-0.0087)
	resistance reversion rate	0.0987	(0.0505-0.1631)
41L			
	$R_{s,\text{before1994}}$	2.6184	(2.3361-2.9074)
	$R_{s,1994-2001}$	0.5251	(0.4403-0.6178)
	$R_{s,2001-2008}$	0.9373	(0.8182-1.0627)
	$R_{s,2008-2015}$	0.9991	(0.746-1.255)
	transmission ratio r_λ	0.7768	(0.5706-1.0052)
	resistance evolution rate	0.0121	(0.0081-0.017)
	resistance reversion rate	0.0903	(0.0381-0.1455)
67N			
	$R_{s,\text{before1994}}$	2.8678	(2.4767-3.2527)
	$R_{s,1994-2001}$	0.5306	(0.4335-0.642)
	$R_{s,2001-2008}$	0.9984	(0.8645-1.1194)
	$R_{s,2008-2015}$	0.8702	(0.6191-1.1162)

mutation	parameter	median	95% HPD
	transmission ratio r_λ	0.6819	(0.5-0.8828)
	resistance evolution rate	0.0059	(0.0026-0.01)
	resistance reversion rate	0.0888	(0.0451-0.1413)
70R			
	$R_{s,\text{before1994}}$	3.2026	(2.8077-3.6536)
	$R_{s,1994-2001}$	0.6253	(0.5292-0.7162)
	$R_{s,2001-2008}$	0.7655	(0.626-0.8831)
	$R_{s,2008-2015}$	0.6788	(0.4019-0.9641)
	transmission ratio r_λ	0.6137	(0.4241-0.8315)
	resistance evolution rate	0.0048	(0.0015-0.0088)
	resistance reversion rate	0.1239	(0.0638-0.2014)
90M			
	$R_{s,\text{before1994}}$	2.5241	(2.1226-2.9732)
	$R_{s,1994-2001}$	0.6651	(0.5386-0.8109)
	$R_{s,2001-2008}$	0.9131	(0.7525-1.089)
	$R_{s,2008-2015}$	0.5074	(0.1795-0.9026)
	transmission ratio r_λ	1.6141	(1.1059-2.1688)
	resistance evolution rate	0.0073	(0.003-0.0128)
	resistance evolution rate (post drug usage)	0.0052	(0-0.0221)
	resistance reversion rate	0.0601	(2e-04-0.1444)
	resistance reversion rate (post drug usage)	0.0578	(0-0.1883)

Table 4. Posterior Bayesian estimates of epidemiological parameters per RDMS. Each line corresponds to one parameter. We report the median estimates together with the 95% highest posterior density interval (HPD). The parameter ”resistance evolution/reversion rate” refers to the time period when drug usage was above 1% in Switzerland. The RMDS 138A has additional estimates for the time before the relevant drug usage became significant (”before drug usage”). The 90M RMDS has additional estimates for the time after the relevant drug usage was ceased (”post drug usage”).

3.1 Non-Convergence of 215Y RMDS

Figure 6 shows the traces of the transmission ratio and the resistance reversion rate for the 215Y RMDS. Both parameters ”jump” between two areas of the state space, indicating a bimodality of the posterior distribution. This is likely due to a lack of data in the resistant deme. The 28 resistant samples fall into 25 distinct cluster. In the three clusters that have two (rather than one) resistant samples, they fall into different parts of the respective trees.

Since the conflict between the two states was not resolved after running the MCMC for more than 2.400 million steps, the results are not reliable and were hence not included.

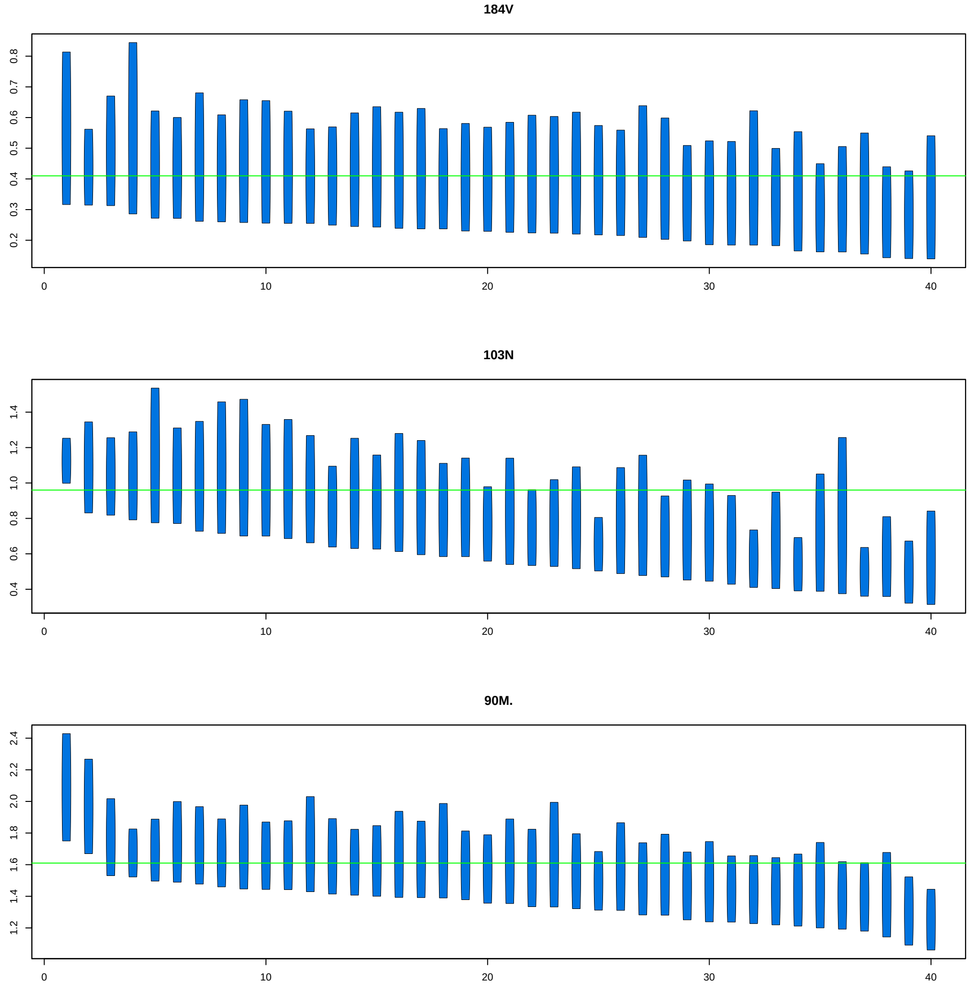

Fig 5. The 95% highest posterior density (HPD) intervals of the reconstructed transmission ratio r_λ for each of the 40 replicates (each blue bar corresponds to one simulation replicate) of the 184V-like simulation set (top), the 103N-like set (middle) and the 90M-like simulation set (bottom). The true value is depicted by a green line.

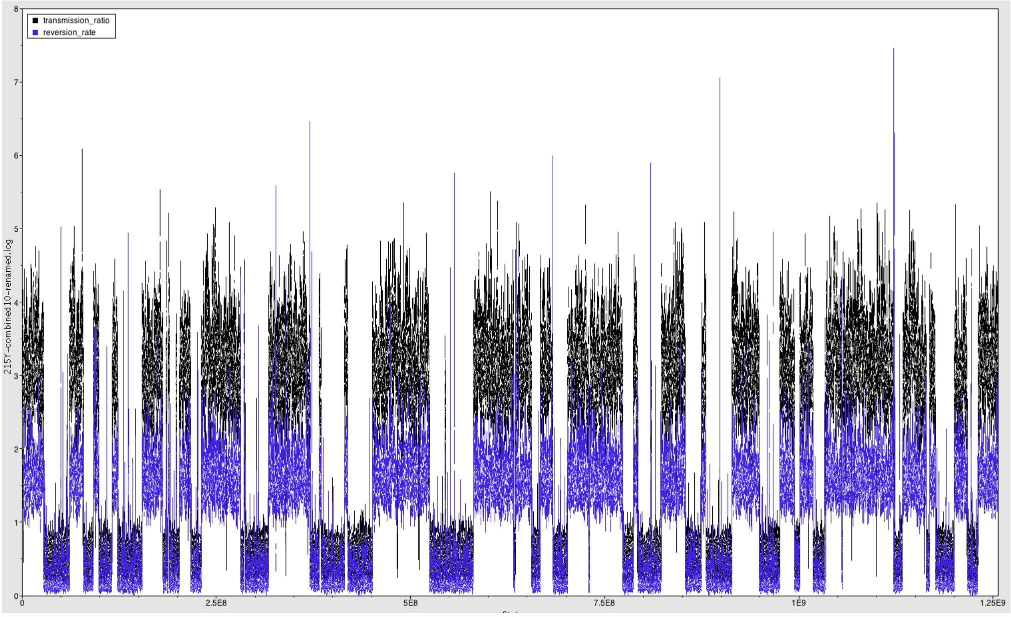

Fig 6. Posterior traces of the transmission ratio (black) and the resistance reversion rate (blue) for the 215Y RMDS, indicating a bimodality of the posterior distribution
